# Recognizing the Vulnerable: Perspectives, Attitudes, and Interests of Women With Uterine Factor Infertility Towards Uterus Allotransplantation

**DOI:** 10.7759/cureus.18891

**Published:** 2021-10-19

**Authors:** Helen Xun, Darya Fadavi, Halley Darrach, Nicole Fischer, Pooja Yesantharao, Franca Kraenzlin, Amanda Nickles Fader, James H Segars, Justin M Sacks

**Affiliations:** 1 Department of Plastic and Reconstructive Surgery, Johns Hopkins University School of Medicine, Baltimore, USA; 2 Department of Obstetrics and Gynecology, Johns Hopkins University School of Medicine, Baltimore, USA; 3 Department of Plastic and Reconstructive Surgery, Washington University School of Medicine, St. Louis Children's Hospital, St. Louis, USA

**Keywords:** uterus allotransplantation, uterine factor infertility, mayer–rokitansky–küster–hauser syndrome, fertility, plastic surgery, transplant, reproductive health, survey, live birth, reproduction

## Abstract

Background: Uterine allotransplantation (UTx) is a novel therapy to allow women with uterine factor infertility (UFI) to bear their own children. To date, over 60 UTx have been performed, resulting in 15 live births. Our study investigates the attitudes, perspectives, and interests of women with UFI towards UTx.

Methods: Anonymous questionnaires were distributed electronically to women diagnosed with UFI at Johns Hopkins Hospital between the years 2003 and 2018.

Results: Thirty-one women with UFI were identified, resulting in 10 completed surveys. The average age was 31.7 ± 6.31 years, and the average age of diagnosis was 20 years (range 14-31); all 10 surveyed women had congenital UFI. Of note, 80% of women agreed that UTx should be an option for women with UFI, and 90% would consider receiving a UTx. The majority of the nine (90%) women who had previously heard of UTx learned about it from the news (5, 50%). When asked to rank the risks related to UTx in order of personal importance, only two women ranked themselves most important; the other woman ranked fetus and donor as more important. All women had health insurance (70% had private insurance), and 90% believed that UTx should be covered by health insurance.

Conclusions: We surveyed women with UFI and found that the majority are willing to have UTx, despite the associated risks of the procedure. Taking into consideration the responses for ranking the importance of risks of the procedure, women with UFI should be considered a vulnerable population, requiring special considerations for UTx informed consents.

## Introduction

It is estimated that 15% of the female reproductive population is infertile, and around 5-10% of these cases are due to uterine dysfunction [1]. Uterine factor infertility (UFI) can be defined as the inability to conceive or maintain pregnancy due to the absence of a uterus, or an anatomically or physiologically dysfunctional uterus. UFI can be due to either an acquired cause (such as hysterectomy [2]) or a congenital malformation (such as Mayer-Rokitansky-Küster-Hauser syndrome [MRKH], affecting one in 4,500 women [3]).

Traditionally, surrogacy and adoption were the only options available to women with UFI desiring children. However, human uterine allotransplantation (UTx) has emerged as a potential therapy to allow women with UFI to bear their own children. Briefly, UTx involves the transplantation of a uterus from a cadaveric or living donor to the recipient. The procedure involves a multidisciplinary team of obstetricians and gynecologists for extended pre-surgical, surgical, and post-surgical care, and transplant surgeons or plastic surgeons for microvascular reconstruction. Prior to transplantation, the recipient undergoes cycles of in vitro fertilization, and viable embryos are implanted following successful transplantation of the uterus. During this time, the recipient must be maintained on immunosuppression. Following the birth of the child via cesarean section, the transplanted uterus is also removed.

In 2014, the first successful human uterine allotransplantation (UTx) clinical trial was completed, introducing UTx as a reality for infertility treatment [4]. That same year, the world’s first case of a live birth following a UTx was performed in a woman with MRKH syndrome in Sweden [5]. In the United States, Cleveland Clinic performed the nation’s first UTx in 2016 [2], and the first live birth following a transplant was reported by Testa et al. at Baylor Dallas in 2017 [6]. To date, there have been over 60 uterine transplantation surgeries reported globally resulting in more than 15 live births [7].

Prior UTx studies have discussed the clinical and surgical aspects surrounding this novel operation [2,4-6]. A number of studies have also explored the ethical [8-10] and psychological [11] concerns involving UTx research and clinical practice. More recently, reports have been published evaluating perceptions toward UTx. Populations evaluated for this have included potential recipients, donors, reproductive endocrinologists, gynecologic surgeons, as well as women from the general population [12-15]. Included in these analyses are reports from Baylor Dallas and the Cleveland Clinic characterizing the women seeking UTx during their respective clinical trials [2,13]. These studies all demonstrated strong interest and support for this procedure from women with infertility, potential donors, physicians, surgeons, and the population in general. In addition, they showed that women with congenital UFI comprise 32-36% of interested candidates [2,13]. These studies also demonstrate that these candidates are now actively seeking UTx clinical trials in the United States. Due to the increasing interest in this procedure, it is imperative now more than ever to optimize study designs and clinical practices for this patient population.

Although previous reports have contributed substantially to the field, studies are still warranted that explore the perspectives of women with congenital UFI regarding UTx as a primary outcome. Our study serves to assess the attitudes, beliefs, and willingness of women with UFI to undergo UTx. We hope these findings will help communicate the perspectives of the UFI community to the medical community and inform clinical and research practices going forward.

## Materials and methods

Patient identification

We designed a cross-sectional study by administering surveys to women with UFI. We identified patients who were seen for UFI at a tertiary academic institute with a reproductive and endocrine infertility service (Johns Hopkins Hospital) from January 1st, 2003 to December 31st, 2018. The patient charts and imaging were used to confirm inclusion criteria of an absence of a functional uterus due to either congenital or acquired reasons, and confirm the patient was English speaking. Patients were excluded if they have received or are under consideration for a UTx. Patients were contacted via telephone or email to participate in the survey. This study was conducted under Johns Hopkins Medicine approval (IRB00172983) and conforms to the Declaration of Helsinki's ethical principles for medical research.

Survey

A Qualtrics (Qualtrics, Provo, Utah) survey was created and distributed to identified UFI patients (Appendix A). Demographics of the patients and their UFI diagnosis, and opinions of uterus transplantation and family planning were collected. Surveys were anonymous and included education about the current state of uterus transplantation.

Data analysis

All statistical analyses were completed using Stata MP, version 13.0 (StataCorp, College Station, Texas). Survey data were visually represented using diverging stacked bar charts.

## Results

Electronic surveys were distributed to 31 individuals, of which 13 were started, and 10 were fully completed. The three incomplete surveys were not analyzed. The average participant age was 31.7 years (range 22-42). Four (40%) surveyors identified as White, one (10%) as Black, two (20%) as Asian, and two (20%) as other, with one of the “other” identified as White, Native American, and Asian (Table 1).

**Table 1:** Summary of demographics and patient characteristics of the survey respondents * One surveyor identified as White, Native American, and Asian. UFI, uterine factor infertility.

No. of completed surveys	10
Age, years	
Mean	31.7 ± 6.31
Range	22 to 42
Cause of UFI	
Congenital UFI	10 (100%)
Age of diagnosis	
Mean	20 ± 4.7
Range	14 to 31
Race, no. of patients	
White	4 (40%)
Black	1 (10%)
Asian	2 (20%)
Other	2 (20%)*
Height (inches)	
Mean	65 ± 2.6
Range	60 to 69
Weight (pounds)	
Mean	155 ± 27.1
Range	130 to 200
Religion	
Christian (non-Catholic)	4 (40%)
Jewish	2 (20%)
Other	2 (20%)
Decline to state	2 (20%)
Highest degree	
High school graduate	1 (10%)
Associate degree	3 (30%)
Bachelor’s degree	3 (30%)
Master’s degree	3 (30%)
Employment status	
Employed	6 (60%)
Military	1 (10%)
Student	1 (10%)
Unemployed	2 (20%)
Yearly household income	
>$150,000	3 (30%)
$100,000 to $149,999	2 (20%)
$70,000 to $79,999	1 (10%)
$50,000 to $59,999	1 (10%)
$30,000 to $39,999	2 (20%)
Declined to respond	1 (10%)
Have insurance	10 (100%)
Private Insurance	7 (70%)
Tricare	1 (10%)
Blue Cross Blue Shield	1 (10%)
Meritain	1 (10%)
Marital status	
Married or domestic partnership	6 (60%)
Divorced	1 (10%)
Single	3 (30%)
Have children	
Yes	2 (20%)
No	8 (80%)

All survey respondents reported graduating high school. Three (30%) had a master’s degree, 3 (30%) had a bachelor’s degree, and three (30%) had an associate's degree. Two reported being unemployed, six (60%) were employed, one (1%) was in the military, and one (1%) was a student. Occupations reported included software engineer, office manager, apprentice estimator, inpatient medical coder, and production. A majority of the surveyors reported being married or in a domestic partnership (6, 60%), one (10%) was divorced, and three (30%) were single.

The mode yearly household income was >$150,000 (3, 30%), followed by two individuals (20%) reporting $100,000 to $149,999, one (10%) reported $70,000 to $79,999, one (10%) reported $50,000 to $59,999, two (20%) reported $30,000 to $39,999, and one (10%) declined to respond. All surveyors had health insurance, most commonly private insurance (7, 70%), including Tricare, Blue Cross Blue Shield, and Meritain (1, 10% each).

All surveyors had congenital UFI (10, 100%), with a mean age of diagnosis at 20 years (range 14 to 31). At the time of the survey, two (20%) had children, and eight (80%) did not have children. When asked if they want to have a child or more children, seven (70%) strongly agreed, and three (30%) were neutral. Considering the options of adoption or surrogacy, more women preferred adoption (70% strongly agree) compared to surrogacy (50% strongly agree). Six (60%) women strongly agreed that they would prefer to give birth to their own child if possible, and the remaining three (30%) were neutral.

Regarding knowledge of UTx prior to this study, one individual reported “not knowledgeable at all,” while the remaining reported varying levels of extremely, very, moderately, or slightly knowledgeable (1, 3, 2, and 3, respectively). When asked where they had heard of UTx, the most commonly reported source was the news (5, 50%), followed by personal research (4, 40%), physician or healthcare provider (2, 20%), social media (2, 20%), and friends/family (1, 10%).

**Figure 1:**
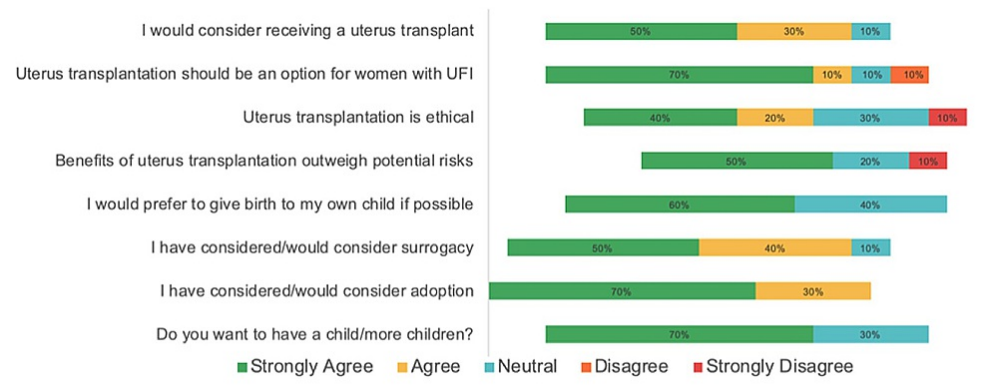
Survey results based on responses of women with uterine factor infertility on uterus transplantation, and considerations for children in the future. UFI, uterine factor infertility.

When asked about their level of agreement with the statement “I would consider receiving a UTx,” the majority of women agreed (5, 50%: strongly agreed; 3, 30%: agreed; 1, 10%: neutral) (Figure 1). More women agreed that UTx should be an option for women with UFI than not (7, 70%: strongly agreed; 1, 10%: agreed; 1, 10%: neutral: 1, 10% disagreed). Half (50%) of the women believed that UTx benefits outweigh potential risks, two (20%) were neutral, and one (10%) strongly disagreed. Five women opted to rank the risks of UTx to self, fetus/developing child, and donor in the order of most importance. Two women ranked the risk of UTx to self as most important, while two ranked the risk to self as the least important. No women ranked the risk to the fetus as the least important, two (40%) ranked the risk to the fetus as most important, and three (60%) ranked the risk to the fetus as second most important. Most women ranked the risk to the donor as least important (3, 60%). All ten women responded when asked if UTx is ethical: 40% strongly agreed, 20% agreed, 30% were neutral, and 10% strongly disagreed. The majority of the women (9, 90%) agreed that UTx should be covered by health insurance.

Lastly, when women were asked if there was any other information they would like to provide, we received two responses in the free form box. The first said: “I have currently been doing research on my own, and have a donor (my mother). If there is any more research that either of us can help in, we are both very interested. Thank you for your time in this survey, I appreciate everyone who is putting knowledge into this for us.” The second said: “[If] I did not already have a child, I would consider a transplant. Now that I have a child, the risk is too great to leave him without a parent.”

## Discussion

The purpose of our study was to identify the attitudes, perspectives, and motivation of women with UFI toward UTx, a novel method allowing women with UFI to bear their own children. We also hoped to provide a voice for the UFI community, ensuring a direct line of communication between the potential recipients of this procedure and the scientific community implementing this surgery into clinical practice. Our results confirmed our hypothesis that the UFI community is interested in UTx as a potential option for allowing women with UFI to have children. For the medical community, it is optimistic that 80% of surveyed women either strongly agreed or agreed that they would consider having a UTx, as there would be candidates available for UTx. However, our results do raise a concern of potential discord within the community. While the majority of women agreed that UTx should be an option (70%), one individual disagreed. This discord is further supported by the mixture of responses from the women when asked whether they believed UTx was ethical: 40% strongly agreed, 20% agreed, 30% were neutral, and 10% strongly disagreed. In essence, our study identified a small minority of the UFI community that believed that UTx should not be an option, or that it is unethical. While there has been a recent movement to position UTx as a “more ethical” alternative to surrogacy (which is illegal in some countries such as Sweden), this position has also not been explored within the UFI community itself [16]. Therefore, it is paramount that the minority who do not believe UFI is ethical, nor should be an option for women with UFI, be provided a safe and anonymous forum to voice their concerns. These perspectives are valuable and warrant future research regarding potential reasons for the opposition of UTx by those with UFI, further facilitating communication between the UFI community and the medical community.

Furthermore, we are concerned that the surveyed women tended to rank the risk to the fetus as more important than the risk to self. Previous studies have identified that women undergoing infertility treatments are more likely to pursue treatments with significant risks as they perceive the benefits of these treatments, including femininity and fertility, to outweigh the risks [7]. This psychology of infertility [17] implies that women with UFI are more likely to pursue risky procedures, which increases their chances of harm, and so they should therefore be classified as a vulnerable population. Given the added context of the substantially higher prevalence of psychiatric disorders in women with UFI [17], we encourage healthcare providers to consider specifically addressing these vulnerabilities. In the interest of protecting these women by providing them with informed, autonomous decision-making, we propose that several steps be taken by healthcare providers. First, the informed consent process for patients should be rigorous and provide appropriate education about UTx, including risks and outcomes, and counseling to provide realistic expectations. Secondly, we agree with Taran et al. [18] that candidates for UTx be provided an extensive psychological assessment to inform, understand, and manage expectations.

We were surprised that our surveyors had a significantly higher annual household income (estimated $99,500 ± 44,578.6, mode of >$150,000) and level of education as compared to national averages in the United States of America (national average income of $48,150, p < 0.0001). All women had health insurance, and the majority (9, 90%) of women believed that UTx should be covered by health insurance. The lay public is also in agreement that UTx should be considered along with other competing claims for healthcare services [19]. However, current US healthcare is faced with inadequate access to numerous healthcare services. An argument can thus be made that equitable access to other services, especially non-elective procedures, may need to be achieved before UTx coverage is seen as financially possible [19]. This can potentially create discordance, as there is a vulnerable patient population that strongly desires the procedure (and with more financial means than the average American to achieve it), in a system that does not make the procedure financially accessible. Consequently, there is a need to protect this vulnerable patient population from financial exploitation and promote equal access to the procedure.

As with most new procedures, the trickle of knowledge from medical professionals to laypeople is often diluted by social media and the news. Uterine transplantation is a procedure that requires systemic immunosuppression which can be associated with side effects of infections, organ dysfunction, and malignancies [7]. These are very real risks that this population needs to comprehend and understand. While it is optimistic that most of the women surveyed had heard of UTx (9, 90%), it is concerning that only two women had heard about it from a physician or healthcare provider - most had heard of it from the news (5, 50%), personal research (4, 40%), or social media (2, 20%). The means by which individuals with UFI receive their information about UTx is important. As a vulnerable population, they should receive unbiased, evidence-based data to best inform their decision-making. Consequently, we recommend that physicians that care for women with UFI be prepared to answer questions and distribute educational material.

Upon reflection of our study, we do recognize that our main weakness is the small sample size of 10 completed surveys. However, as UFI is a rare condition, this single-institution investigation provides valuable insight into the perspectives, attitudes, and opinions of women exclusively with congenital UFI toward UTx. We further acknowledge the limitation of including almost entirely close-ended questions in our survey, which may less effectively capture the breadth of ideas and perspectives of this population than an open-ended question format could offer. We also recognize that the women who chose to complete the survey may be more opinionated and motivated, thus may not necessarily represent the opinions of the UFI community as a whole. However, we feel that it is essential to characterize all opinions regarding UTx from the UFI community, including the extreme positions.

The perspectives toward UTx of women with UFI generally align with those of the general US population. Hariton et al. surveyed 1,247 people from the US general population and found that most (78%) supported while 4% (48) opposed the idea of UTx as an alternative to a gestational carrier [15].

Interestingly, respondents with higher yearly incomes and education levels were more likely to deem the procedure ethical, which parallels the results of our financially advantaged cohort. Moreover, almost half (45%) of the national respondents believed UTx should be covered by insurance, and almost a quarter (24%) did not. In essence, the results of this survey suggest that the US population agrees with our cohort with congenital UFI that UTx should be an acceptable alternative to a gestational carrier, is ethical, and should be covered by health insurance.

## Conclusions

In summary, our study investigates the attitudes, opinions, and openness of women with UFI toward UTx. We found that most women would consider a UTx, and believe that UTx should be an option for women with UFI and should be covered by insurance. However, we identified vital concerns prior to transplantation, including the vulnerability of the target patient population that would require diligent evaluation for candidacy and rigorous education for informed consent, as well as financial considerations for equal access to health services. Consequently, we urge the plastic surgery, transplant surgery, and obstetrics and gynecology communities to continue providing evidence-based material for patient education, and continued communication with the UFI community as UTx becomes more widespread and available internationally.

## Appendices

Appendix A

IRB-approved survey distributed to women identified with uterine factor infertility. The survey was distributed via Qualtrics, and data were deidentified.

Human Uterus Allotransplantation: A Survey of Potential Candidates

Thank you for taking the time to participate in this survey. Please answer each question to your best ability. Participation in the survey is completely voluntary and all questions in the survey are optional. All data will be anonymous and will be de-identified for analysis purposes.

Survey introduction: Uterine factor infertility (UFI) is a medical condition in which a woman cannot give birth because they either do not have a uterus or they have an abnormal uterus that does not function properly. Women can be born with uterine factor infertility (congenital) or they can develop it later in life due to a medical condition (acquired). In the United States, adoption and gestational surrogacy (when a “surrogate” woman carries and gives birth to another woman’s child) are the only two current options for women with uterine factor infertility to have children. Uterus transplantation is a surgical procedure in which a healthy uterus is removed from one woman and placed in another woman who has been diagnosed with uterine factor infertility. Human uterus transplantation is the first possible treatment for uterine factor infertility and would enable women with this condition to give birth to their own child. To date, eight children have been born after a woman has received a uterus transplant (seven in Sweden and one in the United States). Although uterus transplantation has proven successful, the procedure is still in the early stages of clinical research. Many large hospitals in the United States and the international community have developed or are developing human uterus transplantation clinical trials.

Below is a summary of the process of uterus transplantation: (1) A woman’s eggs are collected for in vitro fertilization (IVF). IVF is when a woman’s egg and a man’s sperm are combined in a laboratory setting to create a fertilized egg (embryo). (2) Transplantation procedure occurs: the uterus is removed from the donor and placed in the recipient. (3) The patient is given immunosuppressive (anti-rejection) medication to prevent their body from rejecting the new uterus transplant. This will continue as long as the transplant remains in the patient. (4) If the uterus transplant is successful, the fertilized egg created from IVF will be implanted in the woman’s new uterus approximately one year after surgery. (5) Pregnancy occurs and the baby is carried to term. (6) Baby is born via cesarean section (C-section). (7) After one or two successful pregnancies, the uterus transplant will be removed from the patient. Immunosuppressive (anti-rejection) medication will be stopped after the uterus is removed.

Please note: Your completion of the survey or questionnaire will serve as your consent to be in this research study. If you have any questions about this project, please contact Nicole Fischer () and Helen Xun (). IRB: 00172983 Study PI: Justin M. Sacks, MD, MBA.

Start of Block: Demographic Information

· How old are you?

· What is your height? (Expressed in feet and inches - example, 5'4" would enter 5 in box one and 4 in box two)

o Feet

o Inches

· What is your weight? (In pounds)

· Choose one or more races you identify as:

▢ White

▢ Black or African American

▢ Native American

▢ Asian

▢ Native Hawaiian or Pacific Islander

▢ Other

· Do you identify as Hispanic or Latino?

o Yes

o No

· What is your religion or religions?

▢ Atheist/agnostic

▢ Buddhist

▢ Christian (non-Catholic)

▢ Christian (Catholic)

▢ Jewish

▢ Muslim

▢ Other

▢ Decline to state

· What is the highest level of school you have completed or the highest degree you have received?

o Less than high school degree

o High school graduate (high school diploma or equivalent including General Educational Development)

o Some college but no degree

o Vocational school or technical degree

o Associate degree in college (two-year)

o Bachelor's degree in college (four-year)

o Master's degree

o Doctoral degree (PhD)

o Professional degree (JD, MD)

· What is your current employment status?

o Unemployed

o Employed (please specify profession)

o Student

o Retired

o Military

· What is your yearly household income?

· Do you have health insurance?

o Yes

o No

· What type of health insurance do you have?

o Private insurance

o Medicare

o Medicaid

o Tricare

o Other

· What is your marital status?

o Single

o Married or domestic partnership

o Divorced

o Separated

End of Block: Demographic Information

Start of block: Medical history (some questions shown only if the relevant response to the previous question was selected)

· What is the cause of your uterine factor infertility (UFI)?

o Congenital UFI (born without a uterus or born with a condition that causes the uterus not to work)

o Acquired UFI (uterus was removed surgically or uterus became nonfunctional due to another medical condition)

· What is the cause of your acquired UFI?

o Hysterectomy (uterus removed surgically)

o Dysfunctional uterus (uterus is present but cannot have a child) - cause unknown

o Dysfunctional uterus - radiation damage

o Dysfunctional uterus - myoma

o Dysfunctional uterus - adhesions

o Other

· Why did you have your uterus removed?

▢ Gynecological cancer

▢ Pre-cancer

▢ Fibroids

▢ Endometriosis

▢ Chronic pelvic pain

▢ Abnormal bleeding

▢ Removal due to risk of developing cancer

▢ Obstetric complications

▢ Other

· At what age were you diagnosed with UFI?

· Before you developed UFI, did you ever give birth?

o Yes (state what age or ages)

o No

· How many times have you given birth?

End of block: Medical history

Start of block: Attitudes toward parenthood

· Do you have any children?

o Yes (state how many)

o No

· Do you want to have a child (or more children)?

o Yes

o No

o Unsure

· I have considered/would consider adoption in order to have a child

o Strongly agree

o Agree

o Somewhat agree

o Neither agree nor disagree

o Somewhat disagree

o Disagree

o Strongly disagree

· I have considered/would consider gestational surrogacy in order to have a child

o Strongly agree

o Agree

o Somewhat agree

o Neither agree nor disagree

o Somewhat disagree

o Disagree

o Strongly disagree

· If possible, I would prefer to give birth to my own child (instead of adoption or surrogacy)

o Strongly agree

o Agree

o Somewhat agree

o Neither agree nor disagree

o Somewhat disagree

o Disagree

o Strongly disagree

End of block: Attitudes toward parenthood

Start of block: Uterus transplantation

· Prior to this study, how would you describe your knowledge of human uterus transplantation?

o Extremely knowledgeable

o Very knowledgeable

o Moderately knowledgeable

o Slightly knowledgeable

o Not knowledgeable at all

· Where did you first hear about uterus transplantation?

▢ Never heard of it before this survey

▢ News

▢ Social Media

▢ Personal research

▢ Family/friends

▢ Physician/health care provider

▢ Other (if other, please explain)

· After reading the introduction to this study, how would you describe your knowledge of human uterus transplantation?

o Extremely knowledgeable

o Very knowledgeable

o Moderately knowledgeable

o Slightly knowledgeable

o Not knowledgeable at all

· I understand the risks of uterus transplantation

o Strongly agree

o Agree

o Somewhat agree

o Neither agree nor disagree

o Somewhat disagree

o Disagree

o Strongly disagree

· Please rank the following risks related to uterus transplantation in order of personal importance (1 = most important, 3 = least important; please drag and drop options)

o Risks to self

o Risks to fetus/developing child

o Risks to donor

· I understand the benefits of uterus transplantation

o Strongly agree

o Agree

o Somewhat agree

o Neither agree nor disagree

o Somewhat disagree

o Disagree

o Strongly disagree

· I feel that the benefits of uterus transplantation are greater than the potential risks

o Strongly agree

o Agree

o Somewhat agree

o Neither agree nor disagree

o Somewhat disagree

o Disagree

o Strongly disagree

· I believe that uterus transplantation is an ethical procedure

o Strongly agree

o Agree

o Somewhat agree

o Neither agree nor disagree

o Somewhat disagree

o Disagree

o Strongly disagree

· I believe that uterus transplantation should be an option for women with uterine factor infertility

o Strongly agree

o Agree

o Somewhat agree

o Neither agree nor disagree

o Somewhat disagree

o Disagree

o Strongly disagree

· I believe that uterus transplantation should be covered by health insurance

o Yes

o No

o Unsure

· I would consider receiving a uterus transplant

o Strongly agree

o Agree

o Somewhat agree

o Neither agree nor disagree

o Somewhat disagree

o Disagree

o Strongly disagree

· If there is any other information that you would like to share, please do so below:

End of block: Uterus transplantation
